# 

**Published:** 1889-03

**Authors:** 


### MARCH, 1889.

### CONTENTS.

ORIGINAL PAPERS: Page

Hydrate of Chloral: A Therapeutic Study. By JOHN KENT SPENDER, M.D. Lond. ... 1

Dilatation of the Stomach. By J. MICHELL CLARKE, M.A., M.B. M.R.C.P. ... 21

CLINICAL RECORD:

On the Treatment of Acute Inflammations of the Middle Ear. By CHARLES F. PICKERING, F.R.C.S. ... 32

HEALTH-RESORTS:

Bournemouth. By ALFRED J. H. CRESPI, Wimborne ... 36

REVIEWS OF BOOKS ... 44

NOTES ON PREPARATIONS FOR THE SICK ... 56

PERISCOPE ... 60

SCRAPS ... 71

